# Distinct Non-Human Leukocyte Antigen Antibody Signatures Correlate with Endothelial Crossmatch Status in Lung and Renal Transplant Recipients

**DOI:** 10.3390/ijms251910562

**Published:** 2024-09-30

**Authors:** Fahd Alhamdan, Antonio Coppolino, Adil Sheikh, Anna Miele, Stefi Lee, Allison Gasiewski, Peter Brescia, Isabelle Wood, Arvin Venkat, Tany Thaniyavarn, Selvin Jacob, Mohamed Keshk, Stacia Meadowcroft, Mudassir M. Banday, Mohd Moin Khan, Don Hayes, Anil Chandrekar, Hilary Goldberg, Indira Guleria, Nirmal S. Sharma

**Affiliations:** 1 Brigham and Women’s Hospital, Harvard Medical School, Boston, MA 02115, USA; fah4zan@gmail.com (F.A.); acoppolino@bwh.harvard.edu (A.C.); asheikh5@bwh.harvard.edu (A.S.); anna.miele@emory.edu (A.M.); slee203@bwh.harvard.edu (S.L.); nirmalshya@gmail.com (A.G.); nirmalsharmaconsulting@gmail.com (P.B.); iwood@bwh.harvard.edu (I.W.); arvinvenkat@gmail.com (A.V.); tthaniyavarn@bwh.harvard.edu (T.T.); sjacob7@bwh.harvard.edu (S.J.); mkeshk@bwh.harvard.edu (M.K.); smeadowcroft@bwh.harvard.edu (S.M.); mbanday@bwh.harvard.edu (M.M.B.); mmkhan@bwh.harvard.edu (M.M.K.); achandraker@bwh.harvard.edu (A.C.); hjgoldberg@bwh.harvard.edu (H.G.); iguleria@bidmc.harvard.edu (I.G.); 2 VA Boston Medical Center, Boston, MA 02130, USA; 3 Cincinnati Children’s Hospital Medical Center, University of Cincinnati College of Medicine, Cincinnati, OH 45267, USA; don.hayes@cchmc.org

**Keywords:** Non-HLA, Post-transplant Rejection, ECXM, Allograft

## Abstract

Non-HLA antibodies against heterogeneous targets on endothelial cells have been associated with allograft injuries. The endothelial cell crossmatch (ECXM) is used in the detection of non-HLA antibodies but remains non-discriminatory for specific antibody identification. The primary objective of this study was to delineate the specific non-HLA antibody signatures associated with ECXM positivity and to determine the correlation of ECXM status and non-HLA antibody signatures on allograft health. Serum specimens from 25 lung transplant recipients (LTRs) and 13 renal transplant recipients (RTRs) were collected as part of clinical evaluation, and testing for angiotensin II receptor type 1 (AT1R) and donor-specific MHC class I chain-related gene A (MICA) antibodies and ECXM was performed. Remnant sera were tested for non-HLA antibodies using the LABScreen™ Autoantibody (LSAUT) Group 1, 2, and 3 kits (One Lambda, Inc., Los Angeles, CA, USA). In both cohorts, the concordance of AT1R and MICA together or individually with ECXM+ status was poor (<0.7), suggesting the presence of other unaccounted antibodies. Autoantibody profiling revealed three distinct clusters targeting fibrotic products, cytoskeletal proteins, and cell signaling molecules. A comparative analysis of ECXM+ and ECXM− specimens identified nine and five differentially expressed antibodies in the LTR and RTR cohorts, respectively. Employing machine learning techniques (variable importance, feature selection, ROC-AUC), we derived a five-antibody panel (TNFα, collagen V, CXCL11, GDNF, GAPDH) and a two-antibody panel (TNFα, CXCL9) that effectively discriminated between ECXM+ and ECXM− status in the LTR and RTR cohorts, respectively. Distinct antibody signatures were identified in LTR and RTR cohorts that correlated with ECXM+ status and were associated with allograft dysfunction.

## 1. Introduction

Lung transplantation is the only curative treatment for severe end-stage pulmonary diseases, such as interstitial lung disease (ILD), chronic obstructive pulmonary disorder (COPD), pulmonary arterial hypertension, and cystic fibrosis, among others [1]. Despite medical and surgical advances, the post-transplant longevity of lung allografts remains inferior to other solid organ transplants [2]. Lung allograft health can be negatively impacted by immune-related issues, such as acute cellular rejection (ACR) and antibody-mediated rejection (AMR), and non-immune injuries, such as infection and gastroesophageal reflux, which can cumulatively contribute to chronic lung allograft dysfunction (CLAD) [3].

The critical role of human leukocyte antigens (HLA) in organ matching, ACR, and AMR post-transplant is well documented across all solid organ transplants [4]. Pre-transplant HLA antibodies and post-transplant de novo donor-specific antibodies (DSAs) are associated with an elevated risk of AMR and the development of CLAD [5,6]. Notably, the persistence of rejection in HLA-matched transplant recipients has led to the identification of non-HLA antigens in association with cardiac and renal allograft dysfunction [7,8]. Non-HLA antigens are products of allograft-expressed donor genes, with single nucleotide polymorphisms generating polymorphic peptides that are recognized as foreign by the recipient’s immune system. The resulting non-HLA antibodies may target polymorphic antigen epitopes absent in recipients or epitopes of self-antigens unveiled by apoptosis [9]. A seminal work by Terasaki showed that apart from HLA antibodies, a non-HLA antibody-MHC class 1 chain-like gene A (MICA) was significantly associated with allograft dysfunction in renal transplant recipients (RTRs) [10]. Likewise, emerging studies in lung transplant recipients (LTRs) have reported isolated cases of acute rejection [11] and a potential synergistic effect of angiotensin II type 1 receptor (AT1R) antibody and HLA DSAs linked to AMR [12]. Saini et al. conducted a prospective study of 103 LTRs and found that 16% of those who developed chronic rejection had antibodies to self-antigens, such as k-alpha 1 tubulin and collagen V, but no detectable DSAs [13].

Despite these emerging data, the exact role of circulating non-HLA antibodies—whether as a contributor to or a result of allograft dysfunction—remains ambiguous. The injury mechanisms and pathways triggered by endothelial cell (EC) antibodies interacting with endothelial antigens are poorly understood. It is uncertain whether they serve as markers of damage and byproducts of endothelial stress or if they are the primary instigators of inflammation and dysfunction [9,14,15]. Endothelial cell crossmatch (ECXM) testing using primary human aortic ECs and the XM-ONE assay have been employed to ascertain the clinical relevance of non-HLA antibodies, similar to C1q assays, yet supportive data are scant [14,16,17,18,19]. Present detection methods for EC antibodies, such as cell-based assays using ECs, lack broad availability and specificity in identifying endothelial antibody targets [20]. Consequently, even with positive endothelial crossmatch results, the precise contributions and identifications of specific antibodies are elusive.

Our hypothesis posits that a heterogeneous spectrum of antibodies targeting EC antigens coexists and contributes to the pathogenesis of allograft dysfunction. The principal aim of this investigation is to elucidate the specific non-HLA antibody profiles associated with ECXM positivity and to discern the temporal relationship between ECXM status and non-HLA antibody profiles in relation to allograft health. 

## 2. Results

### 2.1. Concordance among AT1R, MICA, and Positive Endothelial Crossmatch Is Limited

**Figure 1:**
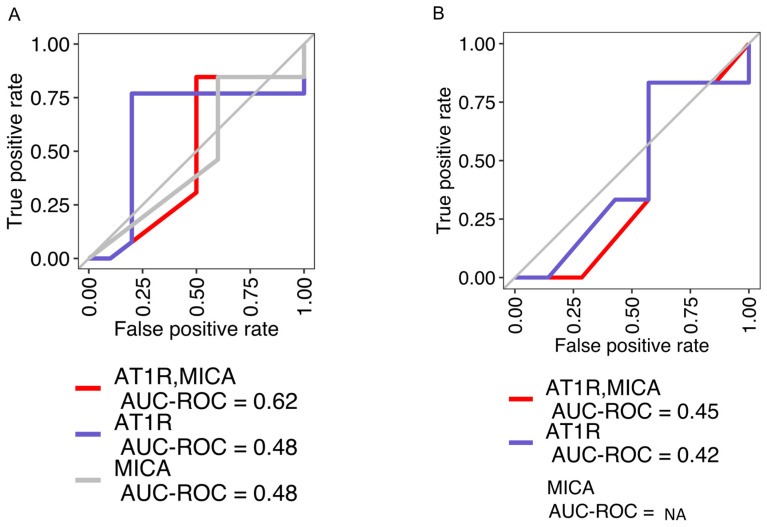
Concordance of AT1R and MICA with EC status in lung transplant recipients (LTRs) and renal transplant recipients (RTRs). ROC-AUC analysis for the classical EC −/+ markers (AT1R and MICA) in (**A**) LTRs and (**B**) RTRs.

Our clinical practice currently utilizes testing for a limited number of non-HLA antibodies, including AT1R and MICA, both targeting endothelial antigens. Additionally, an ECXM is performed to discern the pathogenicity of these non-HLA antibodies [14,19]. First, we investigated whether the presence of AT1R and MICA was correlated with ECXM positivity. Examining AT1R and MICA in the serum specimens from 25 lung transplant recipients (LTRs) and 13 renal transplant recipients (RTRs), a regression analysis demonstrated a low concordance of AT1R (AUC 0.48) and MICA (0.48) with ECXM status in LTRs (Figure 1A)**;** combining both AT1R and MICA in the model marginally improved concordance with ECXM positivity (AUC 0.62). The RTR cohort exhibited even lower AUC scores (Figure 1B). These data suggest that additional, unaccounted antibodies may be contributing to the positivity in the ECXM assays.

### 2.2. Identification of Non-HLA Antibody Clusters in Post-Transplant Patients

**Figure 2:**
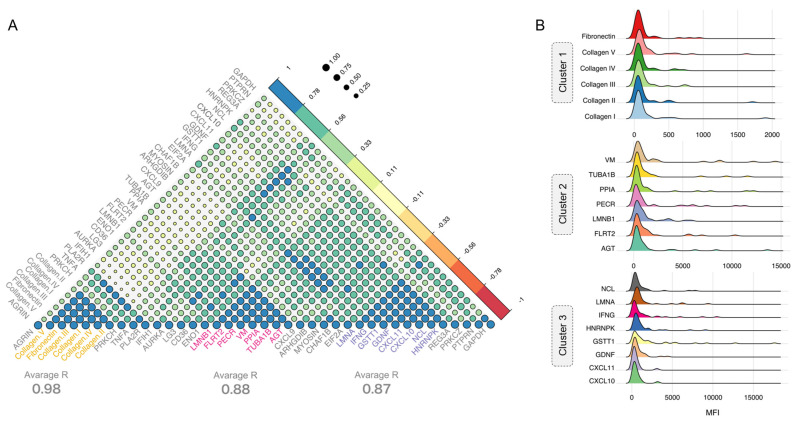
Non-HLA antibodies in post-transplantation. (**A**) Correlation matrix to uncover the similarity pattern among the non-HLA antibodies in the LTR and RTR cohorts. (**B**) Ridge plot depicting the protein levels of the non-HLA antibodies in the three selected clusters.

**Figure 3:**
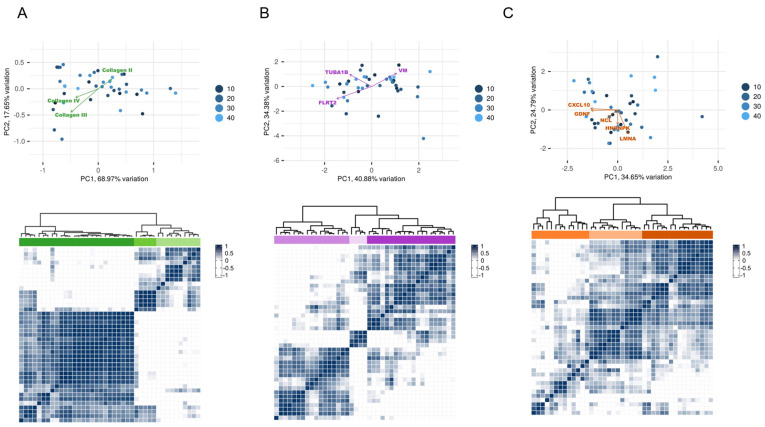
Discrete non-HLA antibody clusters in post-transplant recipients. (**A**–**C**) Principle component analysis (PCA) (**up**) and clustering hierarchal analysis (HCA) (**down**) for the three selected clusters of the non-HLA in the 25 post-transplant LTRs and 10 post-transplant RTRs. The loadings in the PCA represent the strongest factors that drive the distribution of the 38 variables/patients. The HCA showed that the third cluster (**C**) exhibited the most homogeneity among the post-transplant subjects. Different colors: green, purple and orange represent the non-HLA antibody clusters 1, 2 and 3 respectively. Different color shades in the HCA stand for the different patient clusters/groups.

Next, we sought to investigate other unaccounted antibodies that may contribute to ECXM positivity and analyzed matched bio-banked serum samples for autoantibodies from patients with clinical testing performed for AT1R and MICA antibodies. Our assay identified 40 autoantibodies exceeding the detection threshold in the specimens. A subsequent analysis delineated three distinct antibody clusters with robust correlation coefficients (R > 85) in these post-transplant patient specimens of both the LTR and RTR cohorts (Figure 2A). Cluster 1 included antibodies against fibrotic elements, Cluster 2 comprised antibodies targeting cell surface receptor proteins, and Cluster 3 contained antibodies against cell signaling molecules, with their expression levels (Figure 2B). A hierarchical clustering analysis further revealed the predominant contributors within each cluster (Figure 3A–C). For Cluster 1, three anti-collagen antibodies prominently influenced the antibody distribution, while Cluster 2 was influenced by antibodies to vimentin, tubulin, and fibronectin leucine-rich transmembrane protein 2. In Cluster 3, five antibodies to chemokines CXCL10 and four other proteins drove the cluster distribution in the same direction, which gave this cluster the most consistency across the post-transplant samples (Figure 3C Down).

### 2.3. Antibody Subtypes as Predictors for Endothelial Crossmatch Subtypes

**Figure 4:**
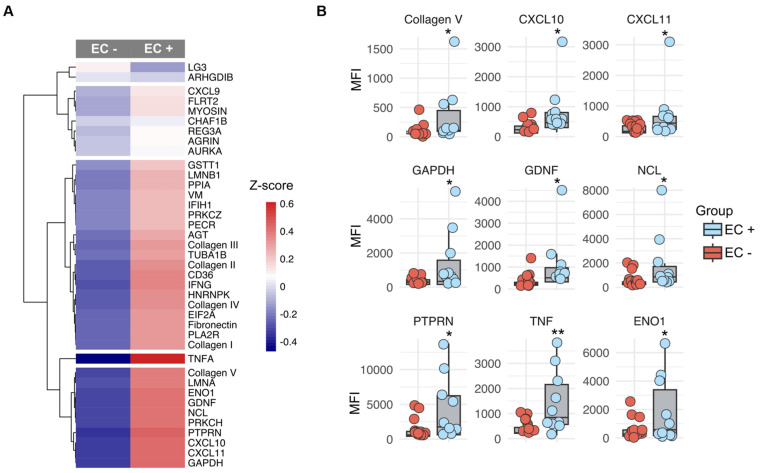
Non-HLA antibody and correlation to EC status in LTRs. (**A**) Z-score analysis for the non-HLA antibodies in the positive and negative crossmatch endothelial cells regardless of significance. (**B**) Nine significant non-HLA antibodies to differentiate between the ECXM+ and ECXM− in LTRs. Data are mean ±SE and were analyzed using a *t*-test. * and ** denote *p* < 0.05 and <0.005, respectively.

**Figure 5:**
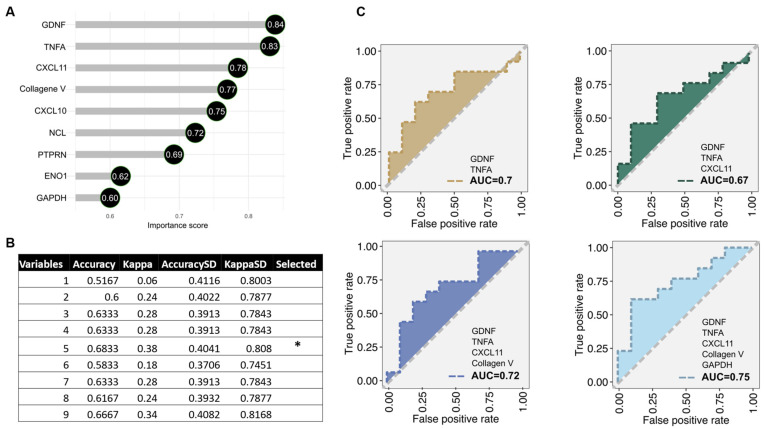
Building a prediction model to differentiate EC status in the LTR cohort utilizing machine learning methods. (**A**) Variable importance analysis implemented with learning vector quantization to probe whether the variables that are important enough (>0.5) to build the prediction model. (**B**) Feature selection analysis involves removing the weakest features/markers and selecting the best combination of markers to represent the prediction model (*) “Collagen V, TNFa, CXCL11, GDNF, and GAPDH”. (**C**) AUC-ROC analysis implemented with random forest to verify the results of the feature selection analysis and address the markers as optimal predictors for EC +/− differentiation.

We evaluated whether a distinct antibody signature can discriminate between ECXM+ and ECXM− status due to the suboptimal prediction by AT1R and MICA antibodies. A z-score analysis of LTR (Figure 4A) and RTR (Supplementary Figure S1A) specimens highlighted several antibodies with varied expressions between the ECXM+ and ECXM− cohorts. Of these, nine non-HLA antibodies against collagen V, TNFa, nucleolin (NCL), CXCL10, CXCL11, glial cell line-derived neurotrophic factor (GDNF), glyceraldehyde 3-phosphate dehydrogenase (GAPDH), receptor-type tyrosine-protein phosphatase-like N (PTPRN), and alpha-enolase (ENO1) were significantly overexpressed in the ECXM+ group compared to ECXM− in the LTR cohort (Figure 4B). Moreover, the RTR cohort exhibited five non-HLA antibodies against collagen I, CXCL9, fibronectin, regenerating family member 3 alpha (REG3A), and TNFa (Supplementary Figure S1B). After affirming that all nine non-HLA antibodies in the LTR cohort exceeded a variable importance score greater than 50% (Figure 5A), a feature selection analysis detected a combination of five non-HLA antibodies to be the optimal discriminators differentiating between ECXM+ and ECXM− (Figure 5B). This signature encompassed antibodies to GDNF, TNFa, CXCL11, collagen V, and GAPDH. Next, we validated the outcome of the feature selection analysis with an ROC-AUC analysis implemented with the random forest machine learning method. By testing an increasing combination of the previous signature according to the variable importance order, the five non-HLA antibodies achieved the highest discriminatory ability (AUC = 0.75) between ECXM+ and ECXM− status for LTR-only specimens (Figure 5C). Similar analyses were applied for the five non-HLA antibodies in the LTR cohort, and two of them (TNFa and CXCL9) achieved AUC = 0.79 (Supplementary Figure S2A–C).

### 2.4. Distinct Antibody Signature Correlates Inversely with Lung Function in LTRs

**Figure 6:**
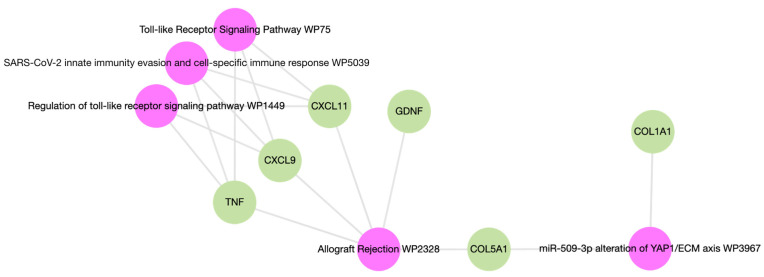
Biological pathways analysis of the non-HLA antibody signature in LTR and RTR cohorts using Wikipathway database.

**Figure 7:**
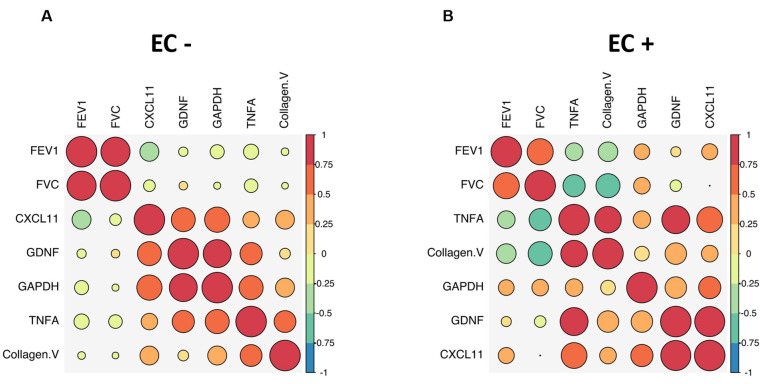
Clinical correlation of non-HLA antibodies with pulmonary function tests in LTRs. Correlation matrix to depict the regression coefficients between TNFα, collagen V, GDNF, GAPDH, and CXCL11 and FEV1 and FVC in (**A**) ECXM− LTR subjects and (**B**) ECXM + LTR subjects. Circle size and color coding in the legend provide the positive and negative correlation strengths.

We conducted a biological pathway analysis of the predictor markers to assess their relevance to allograft dysfunction in both cohorts. A Wikipathway analysis revealed a shared association of collagen V, TNFa, CXCL11, CXCL9, and GDNF with allograft rejection (Figure 6). Next, to evaluate the clinical significance of the autoantibody signature in the LTR cohort, we correlated the identified markers with pulmonary function testing using spirometry. The antibody signature in LTRs (TNFa, collagen V, CXCL 11, GDNF, and GAPDH) correlated inversely with lung function in EC+ subjects (Figure 7A,B). Among the markers, TNFa and collagen V had the strongest negative correlation with both forced vital capacity (FVC) and forced expiratory volume in one second (FEV1) of EC+ subjects.

## 3. Discussion

There is increasing evidence of an important contribution of non-HLA DSAs in solid organ dysfunction after transplantation [8,21,22,23]. The current study establishes a novel non-HLA antibody signature (comprising collagen V, TNFa, CXCL11, GDNF, and GAPDH) in LTRs that strongly aligns with ECXM positivity and inversely correlates with lung allograft function. These findings suggest a potential benefit of widening non-HLA antibody assessment for the clinical evaluation of allograft dysfunction and provide a basis for future in-depth cohort studies aimed at delineating the direct causative links between these non-HLA antibody patterns and lung allograft failure. Furthermore, this research propels the pursuit of developing actionable biomarkers for the timely detection of allograft dysfunction.

A link between MICA and rejection in kidney and heart recipients has previously been reported [21,23]. Further, antibodies directed at the endothelial markers, AT1R and ETAR, are associated with allograft dysfunction [24,25]. Likewise, AT1R and MICA antibodies have been linked to acute allograft and late allograft injury, respectively, in liver transplant recipients [26,27]. Contemporary protocols in lung transplantation at our center involve screening for MICA and AT1R antibodies, accompanied by ECXM testing, to detect antibodies against primary EC lines (avoiding HLA DSA and presumed to be donor-specific)—a key target of the recipient’s immune response [14,16,19]. Despite its use in clinical practice, it remains unclear what the true correlation of AT1R and MICA antibodies is with ECXM positivity. Our study found that both AT1R and MICA alone or in combination have a low correlation with ECXM status in LTRs. We also utilized an RTR cohort as a comparator group in our study and found similarly low concordance of AT1R/MICA with ECXM positivity. These findings suggest the potential contributions to ECXM positivity by other unaccounted antibodies. Further adding strength to this hypothesis is that HLA DSAs were absent in all but one subject at the time of sampling, thereby discounting the role of HLA DSAs in cross-reactivity and ECXM positivity.

To address the question of other unaccounted antibodies, we investigated additional non-HLA antibodies in sera from LTRs. Historically, non-HLA antibodies are thought to occur in response to unrecognized donor epitopes or self-antigens that surface during cellular injury or apoptosis [28], but the mechanism of how the antibodies develop and impact allograft remains poorly understood. Our analyses identified three distinct non-HLA antibody clusters characterized by high association coefficients, indicative of antibodies against profibrotic products, structural cytoskeletal proteins, cell signaling molecules, and key intracellular enzymes. We found a significant upsurge in antibodies against extracellular matrix (ECM) components collagen V, collagen I, and fibronectin in ECXM+ subjects in the LTR cohorts. Collagen V is found both extracellularly and intracellularly and lines the basement membrane of vascular ECs [29,30]. Further, the expression of antibodies against glial cell line-derived neurotrophic factor (GDNF), an intracellular proliferation factor was significantly increased. Further supporting that non-HLA antibody development targets cellular components was our discovery of antibodies against GAPDH and ENO1, both of which are crucial intracellular enzymes in glycolysis and cellular equilibrium [31,32]. We compared these findings in the LTR cohort with an RTR cohort. There were commonalities in the antibody signatures between the two cohorts. The RTR cohort highly expressed antibodies to pro-fibrotic products collagen and fibronectin, as well as antibodies to chemokines and pro-inflammatory cytokines in the ECXM+ group. However, RTR had antibodies to collagen I and CXCL9 versus antibodies to collagen V and CXCL10 and 11 in the LTR cohort. These subtle differences may represent the impact of the local microenvironment in the allograft, such as the presence of higher fibrillar collagen V in the allograft lungs compared to the kidneys [33]. Our collective findings imply that cell damage to endothelial and epithelial cells exposes antigens, thereby generating autoreactive non-HLA antibodies in transplant recipients. We find this interesting as it appears that initially the non-HLA antibody development, at least in part, is the result of allograft injury rather than the cause of it. Nevertheless, once formed, these non-HLA antibodies may contribute to ongoing allograft injury.

Previous work shows that complement activation and natural killer (NK) cell-mediated lysis is associated with non-HLA antibody-induced allograft injury [34,35], leading to cell disruption. Likewise, lymphocyte transfer from collagen V-immunized rats can initiate autoimmunity, implying a more direct role in allograft dysfunction [36,37]. Autoantibodies against collagen V and k-alpha tubulin are associated with chronic lung rejection, while ENO1 and GAPDH antibodies occur in chronic heart rejection [7,38]. Further, ENO1 antibodies have been linked to fibrogenesis in glomerular nephropathy and interstitial lung disease [39,40]. In our cohort, we observed increased expressions of antibodies against TNFa and CXCL11 in the ECXM+ group. Antibodies to the chemokine CXCL11 have been associated with chronic heart, lung, and renal allograft dysfunction [7,41]. In heart recipients with coronary artery vasculopathy, T cells tend to produce less IL-10 and more IL-17 and IFNγ, indicating a Th17 response that promotes autoimmunity [42]. Given that TNFa is secreted by both activated lymphocytes and CXCL11 is a chemokine induced by IFNγ that attracts activated T cells and NK cells, it is plausible that antibodies to these chemokines are a consequence of their elevated expression during allograft injury [7,43]. These findings suggest that transplanted solid organs may represent a microenvironment supportive of non-HLA antibody development across a variety of mechanisms. It is conceivable that these self-reactive non-HLA antibodies are generated against structural proteins and cellular enzymes that initiate a feedforward cycle of endothelial damage, ECM disintegration, inflammation, and the disruption of cellular processes. The result is continuous repair and remodeling that ultimately leads to allograft dysfunction. Therefore, the current study reinforces the need for further investigations into non-HLA antibody development before and after transplantation as there may be factors before the transplant that predispose some recipients to develop autoimmunity.

Supporting our concerns regarding autoimmunity, recent studies suggest that treatment of circulating non-HLA antibodies could be beneficial, particularly when allograft dysfunction or AMR is present [22,44]. However, the effectiveness of ECXM positivity as an indicator of significant non-HLA antibodies—akin to the role of C1q assays for HLA antibodies—remains uncertain. It is important to note that ECXM positivity has not been established as a factor in negative clinical outcomes for transplant recipients. A particular challenge with conventional ECXM (XM-ONE) lies in its use of precursor ECs as a test substrate, which might not display all potential antigens of transplanted tissue [15]. Furthermore, the use of the primary EC line and XM-ONE is limited to detecting only surface proteins, potentially overlooking antibodies that target crucial integral membrane, nuclear, cytoplasmic, or matrix antigens [15]. To address these challenges, we explored distinctive antibody signatures targeting antibodies to TNFa, collagen V, CXCL11, GDNF, and GAPDH, which robustly distinguished between ECXM+ and ECXM− status factored in intracellular antigens, in addition to surface markers. Importantly, our pathway analysis indicated an interconnection of these specific non-HLA antibodies and a pivotal role in allograft dysfunction with an inverse correlation to lung function with spirometric measurements in the LTR cohort. We believe these results warrant a prospective study to further explore the larger impact of non-HLA antibodies on allograft function and develop predictive models that may assist in clinical decision-making.

The current study suffers from inherent constraints, primarily due to its observational design and the limitation of being a single-center cohort study with a small study population. Despite these limitations, the inclusion of lung and kidney transplant recipients addresses some of these restrictions and strengthens our findings, which suggest a potential systemic influence of non-HLA antibodies in solid organ recipients. Further, it is unclear whether high levels of non-HLA antibodies in asymptomatic subjects are concerning and should be treated or not. It is certainly possible that non-HLA autoantibodies, similar to HLA antibodies, may not act in isolation, and other factors, such as immune tolerance, genetic predispositions, or concurrent immunomodulatory therapies could alter the expected pathogenicity. Likewise, the non-HLA assay’s preset cutoffs (as defined by OL) provide a useful guideline, but they may not capture all clinically relevant cases. Thus, additional parameters, including clinical correlation and longitudinal monitoring, may be required to robustly interpret results that are outside these predefined thresholds. Specific to LTRs, we observed significant correlations between non-HLA antibodies, ECXM+ status, and a decline in lung allograft function. However, our findings should be taken with caution due to the cross-sectional nature of our small cohort until multi-center studies can be performed to further validate our preliminary findings. Importantly, our results are hypothesis-generating and serve as a foundation for future research, with longitudinal specimen analyses, including a pre-transplant non-HLA repertoire, to confirm the temporal sequence and establish a cause-and-effect relationship between non-HLA antibodies and allograft dysfunction.

In summary, the current study identified a unique antibody profile that is associated with ECXM+ status and allograft dysfunction. As research in this field continues, an enhanced understanding of non-HLA antibodies in the context of allograft dysfunction will not only improve our understanding of their role in autoimmunity but may assist in the development of biomarkers of disease and novel pharmaceutical targets to improve allograft function.

## 4. Materials and Methods

### 4.1. Human Sample Collection

Serum specimens from LTRs (*n* = 25) and RTRs (*n* = 10) were collected as part of investigations for a decline in lung function and renal function, respectively. AT1R, MICA antibody testing, and ECXM were performed for clinical evaluation. Remanent serum specimens were bio-banked at −80 °C. Demographic details are provided in Table 1. Pulmonary function tests were abstracted for LTRs at the time of sample acquisition (T0) and one-year post-sample acquisition (T2). Likewise, bio-banked serum specimens from a cohort of renal transplant recipients (RTRs) who underwent AT1R, MICA, and ECXM testing for clinical indications were also evaluated. 

### 4.2. Sample Processing and Autoantibody Testing

Sera from LTRs and RTRs were tested for non-HLA antibodies using the LABScreen™ Autoantibody (LSAUT) Group 1, 2, and 3 kits (One Lambda, Inc., Table 1 and Table 2), according to manufacturer’s instructions [1]. Groups 1 and 2 were tested simultaneously in the same well for each sample, while Group 3 was tested separately. All sera were centrifuged prior to testing. The kit’s negative and positive controls were tested when applicable. For the combined Group 1 and 2 tests, 40 µL of serum was combined with 5 µL of each Group 1 and Group 2 bead. For the Group 3 tests, 20 µL of serum was combined with 5 µL of Group 3 beads in a separate well. Trays were incubated at 20–25 °C for 30 min in the dark with shaking then washed three times using the spin and flick method [1]. For LSAUT1 and LSAUT2 assays (Table 2), LABScreen wash buffer (Cat. #LSPWABUF) was used, while Group 3 wash buffer (Cat. #LSPAUT-3) was used for LSAUT3 assays (Table 3). Wash buffers were diluted with distilled water to make a 1× wash solution. After the three washes, 100 µL of diluted 100× PE-conjugated anti-human IgG (1:100 dilution) was added to each well and incubated at 20–25 °C for 30 min in the dark with shaking. After incubation, trays were centrifuged, and the supernatant was removed by flicking and blotting. Two additional washes were performed as previously described. The beads were resuspended in 80 µL of PBS, mixed well, and then analyzed on the Luminex LABScan3D (VH Bio, Gateshead, UK) using the One Lambda templates. 

### 4.3. Quality Control and Analyses

The results for samples with a high background (NC > 1500), bead failures (count < 50), or a low PC (<500) were deemed invalid, and repeat testing was performed. For samples with low bead counts on LSAUT3 (<50), extended high-speed centrifugation (15,000× *g* for 30 min) was performed on these sera before repeat testing. The output.csv files were obtained from the analyzer and transferred into Fusion Research 6.2 Software for analysis. To determine positivity, results were analyzed using a 95% cutoff. Any additional targets that reacted positively at the 75% and 85% cutoffs were noted as weakly positive in the comment section. Raw and normalized (S#N-SNC bead) MFI values were extracted from the Fusion software for statistical analysis.

### 4.4. Statistical Analysis

Similarity pattern and clustering detection of the non-HLA antibodies was performed using the R package (corrplot v0.92) with the hclust method. A principal component analysis (PCA) was utilized to define the distribution of the post-transplant samples for each of the three clusters with their potential loadings using the iDEP v1.0 tool. For the hierarchal clustering analysis (HCA), Spearman as the correlation coefficient and Euclidean as the distance matrix computation method were performed. Heatmaps were generated with the R package (pheatmap v1.0.12). For building the prediction model, the following three machine learning methods were used: 1. variable importance was implemented with the learning vector quantization algorithm using R packages (caret v6.0.94) and (mblench v2.1.3); 2. feature selection implemented with the cross-validation method was performed via the same previous packages; 3. receiver operating characteristic-area under the curve (ROC-AUC) combined with the random forest machine learning method was conducted using R packages (caret v6.0.94) and (MLeval v0.3). For testing differences between ECXM+ and ECXM−, a *t*-test was used for continuous variables. Likewise, FEV1 and FVC change were used as continuous variables for all correlations. R packages were run on R software version 4.2.2 (31 October 2022) “Innocent and Trusting”.

## Figures and Tables

**Table 1:** Demographics (lung transplant recipients).

	ECXM+ (*n* = 11)	ECXM− (*n* = 14)
Age-years (Mean (SD))	53.8 (13.73)	57.5 (10.05)
Gender (% male)	36%	64%
FEV1 (L) Mean (SD)	1.58 (0.59)	1.64 (0.82)
FVC (L) Mean (SD)	2.38 (0.58)	2.38 (0.60)
Duration from transplant (Mean (SD))	1725 (2389)	1355(2086)
Immunosuppression		
T, M, P	54.5%	35.7%
T, A, P	36.3%	21.4%
T, S, P	0%	7%
T, P	9%	3.7%

Abbreviations: M: mycophenolate, P: prednisone, A: azathioprine, S: sirolimus, T: tacrolimus.

**Table 2:** Non-HLA antibodies tested using LABScreen™ Autoantibody Group 1 (LSAUT1) and 2 (LSAUT2) kits (One Lambda, Inc.). To determine antibody positivity, a 95% cutoff was on HLA Fusion Research Software 6.2. This value is shown in terms of mean fluorescence intensity per lot tested.

LSAUT Kit	Lot	Lot
LSAUT1	002	007
LSAUT2	002	003
Specificity	95% Cutoff MFI	95% Cutoff MFI
ENO1	4218	4218
FLRT2	688	688
VM	820	820
TUBA1B	1987	1987
CD36	1591	1591
IFIH1	3870	3870
MYOSIN	9341	9341
AGT	1641	1641
PTPRN	3042	3042
AURKA	4892	4892
CHAF1B	11,722	11,722
PPIA	3292	3292
EIF2A	6901	6901
GSTT1	6136	6136
LMNA	6633	6633
PRKCZ	9104	9104
PECR	4120	4120
PRKCH	1048	1048
LMNB	2065	2065
CXCL11	309	309
CXCL10	285	285
CXCL9	575	575
AGRN	504	504
ARHGDIB	3918	3918
GDNF	1004	1004
HNRNPK	845	845
IFNG	498	498
NCL	3669	3669
REG3A	86	86
GAPDH	508	508
TNFA	5331	5331
PLA2R	195	195
LG3	3801	4154

ENO1, alpha-enolase; FLRT2, fibronectin leucine-rich transmembrane protein 2; VM, vimentin; TUBA1B, tubulin alpha-1B chain; CD36, platelet glycoprotein 4; IFIH1, interferon-induced helicase C domain-containing protein 1; MYOSIN, myosin-binding protein C, cardiac-type; AGT, angiotensinogen; PTPRN, receptor-type tyrosine-protein phosphatase-like N; AURKA, aurora kinase A-interacting protein; CHAF1B, chromatin assembly factor 1 subunit B; PPIA, peptidylprolyl cis-trans isomerase A; EIF2A, eukaryotic translation initiation factor 2A; GSTT1, glutathione S-transferase theta-1; LMNA, prelamin A/C; PRKCZ, protein kinase C zeta; PECR, peroxisomal trans-2-enoyl-CoA reductase; PRKCH, protein kinase C eta; LMNB, lamin B1; CXCL11, C-X-C motif chemokine 11; CXCL10, C-X-C motif chemokine 10; CXCL9, C-X-C motif chemokine 9; AGRN, agrin; ARHGDIB, Rho GDP-dissociation inhibitor 2; GDNF, glial cell line-derived neurotrophic factor; HNRNPK, heterogeneous nuclear ribonucleoprotein K; IFNG, interferon gamma; NCL, nucleolin; REG3A, regenerating islet-derived protein 3 alpha; GAPDH, glyceraldehyde 3-phosphate dehydrogenase; TNFA, tumor necrosis factor alpha; PLA2R, secretory phospholipase A2 receptor; LG3, perlecan.

**Table 3:** Non-HLA antibodies tested using LABScreen™ Autoantibody Group 3 (LSAUT3) kits (One Lambda, Inc.)**.** To determine antibody positivity, a 95% cutoff was on HLA Fusion Research Software 6.2. This value is shown in terms of mean fluorescence intensity per lot tested.

LSAUT Kit	Lot	Lot	Lot
LSAUT3	002	004	005
Specificity	95% Cutoff MFI	95% Cutoff MFI	95% Cutoff MFI
Collagen I	375	375	375
Collagen II	155	155	155
Collagen III	128	128	128
Collagen IV	71	71	71
Collagen V	187	187	187
Fibronectin	141	141	141

## Data Availability

Raw data will be available on reasonable request from the corresponding author.

## Supplementary Materials

The supporting information can be downloaded at https://www.mdpi.com/article/10.3390/ijms251910562/s1.
