# Supplementary material for: Distinct Non-Human Leukocyte Antigen Antibody Signatures Correlate with Endothelial Crossmatch Status in Lung and Renal Transplant Recipients
Source: Int J Mol Sci. 2024 Sep 30;25(19):10562. doi: 10.3390/ijms251910562 (PMC11476851; doi:10.3390/ijms251910562)
Supplement: Supplementary file 1 [file ijms-25-10562-s001.zip › ijms-3157601-supplementary.pdf]

Supplementary Material (Figures and Table)

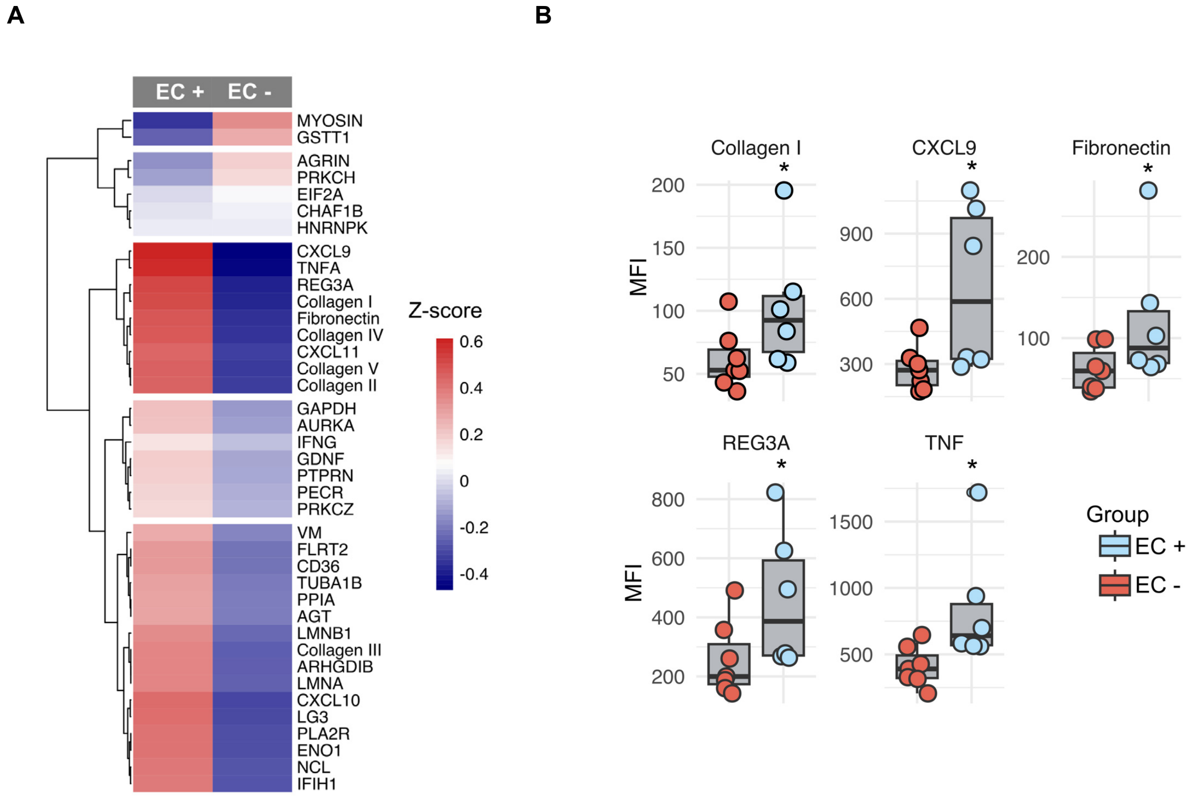

Supplementary Figure S1. Non-HLA antibody and correlation to EC status in RTRs. A) Z score analysis for the non-HLA antibodies in the positive and negative crossmatch endothelial cells regardless of significance. **B)** Five significant non-HLA antibodies to differentiate between the ECXM + and ECXM – in RTRs. Data are mean ± SE and were analyzed using t-test.

A

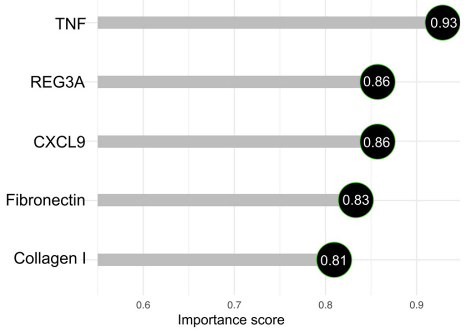

B

Variables	Accuracy	Kappa	AccuracySD	KappaSD	Selected
1	0.6389	0.3333	0.4271	0.8165	
2	0.8056	0.6667	0.3058	0.5164	*
3	0.8056	0.6667	0.3058	0.5164	
4	0.7222	0.5	0.4431	0.8367	
5	0.7222	0.5	0.4431	0.8367	

C

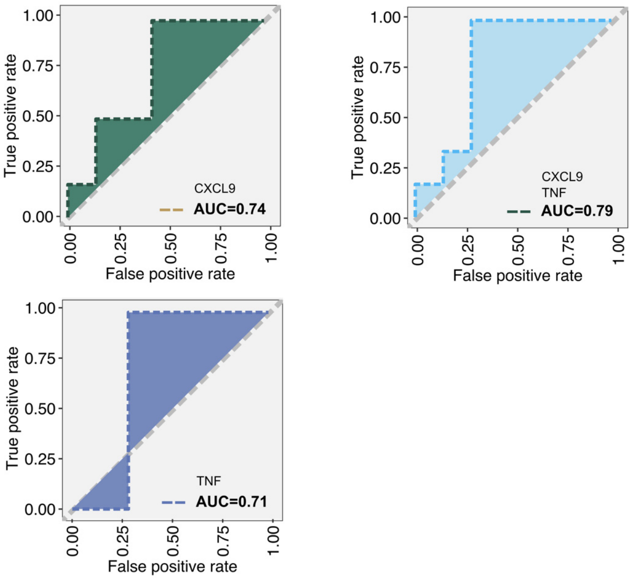

Supplementary Figure S2. Building a prediction model to differentiate EC status in the RTR cohort utilizing machine learning methods. A) Variable Importance analysis implemented with Learning Vector Quantization to probe whether the variables are important enough (>0.5) to build the prediction model. **B)** Feature selection analysis involves removing the weakest features/markers and selecting the best combination of markers to represent the prediction model (*) “CXCL9 and TNFa”. **C)** AUC-ROC analysis implemented with Random Forest to verify the results of the feature selection analysis and address the markers as optimal predictors for EC +/- differentiation.

Supplementary Table S1 (Lung transplant recipients)

Pt No	Age	Sex	Race	Pretx dx	Sample date after transplantation (Days)	AT1R	MICA	ECXM status	HLA ab
1	62	M	White	CPFE	783	-	+	+	CL1-, CL2-
2	49	M	White	ILD	527	+	-	+	CL1-, CL2-
3	59	F	American Indian	COPD	901	-	-	+	CL1-, CL2+(DR51), Non DSA
4	34	F	Black	Bronchiectasis	17	+	+	+	CL1-, CL2-
5	65	F	White	Polymyositis and ILD	448	+	-	+	CL1+(B51), CL2+(DQ7) DSA
6	67	M	White	IPF	1690	+	-	+	CL1-, CL2-
7	65	M	Whitev	Hypersensitivity pneumonitis	953	+	-	+	CL1-, CL2-
8	67	F	White	IPF	1540	-	+	+	CL1-, CL2-
9	28	F	White	Bronchiolitis obliterans	697	-	-	+	CL1-, CL2-
10	43	F	White	Lymphangioleiomyomatosis	3105	-	+	+	CL1-, CL2+(DQ7), Non DSA
11	51	F	White	IPF	697	+	-	+	CL1-, CL2-
12	66	M	White	IPF	510	-	-	-	CL1-, CL2-
13	70	M	White	COPD	1613	-	-	-	CL1-, CL2-
14	39	F	White	IPAH	364	-	-	-	CL1-, CL2-
15	54	M	White	idiopathic pulmonary fibrosis	102	-	+	-	CL1-, CL2-
16	63	F	White	Hypersensitivity Pneumonitis	387	+	-	-	CL1-, CL2+, Non DSA
17	57	M	Unknown	Dermatomyositis and ILD	819	-	-	-	CL1-, CL2-
18	41	F	White	Cystic fibrosis	337	-	+	-	CL1+, CL2+ (DR7), Non DSA
19	57	M	White	Idiopathic pulmonary fibrosis	1232	-	-	-	CL1-, CL2+ (DR103, DR9, DR10), Non DSA
20	70	M	White	ILD	207	-	-	-	CL1-, CL2-
21	66	M	White	IPF		-	-	-	CL1-, CL2-
22	47	F	White	Pulmonary Fibrosis	2620	-	-	-	CL1+, CL2+, Non DSA
23	62	M	White	COPD		-	-	-	CL1-, CL2-
24	52	F	Asian	Lymphangioleiomyomatosis	3881	-	-	-	CL1-, CL2-
25	62	M	White	Pulmonary Fibrosis	525	+	-	-	CL1-, CL2-

IPAH: idiopathic pulmonary arterial hypertension, CPFE: Combined pulmonary fibrosis and emphysema, IPF: idiopathic pulmonary fibrosis.

Supplementary Table S2 (Renal transplant recipients)

Pt No	Age (Y)	Sex	Race	Pretx dx	Sample date after transplantation (days)	AT1R	MICA	ECXM status	HLA ab
1	46	F	Hispanic	Hypertension	4393	-	-	+	CL1+, CL2+, Non DSA
2	35	M	Black	IgA nephropathy	2061	+	-	-	CL1+ (A34), CL2-, Non DSA
3	71	M	White	Focal glomerular sclerosis	2653	-	+	-	CL1-, CL2-
4	33	M	White	Focal glomerular sclerosis	249	-	-	-	CL1-, CL2+ (DQ7, DQ2), Non DSA
5	51	F	White	Polycystic kidneys	4214	-	-	+	CL1+ POS, CL2- Non DSA
6	78	F	White	Hypertension	504	+	-	-	CL1-, CL2-
7	55	M	Black	Hypertension	1383	-	-	-	CL1+ (A2), CL2- CL1-, Non DSA
8	43	M	Black	Shock/tubular necrosis from viral myocarditis	2052	+	-	-	CL1+ (A11), CL2- Non DSA
9	50	M	White	IgA nephropathy	360	-	-	+	CL1-, CL2+, Non DSA
10	35	F	White	Hypertension	3279	-	-	-	CL1-, CL2-
